# Accounting for Intraindividual Profiles in the Wechsler Intelligence Scales Improves the Prediction of School Performance

**DOI:** 10.3390/children9111635

**Published:** 2022-10-27

**Authors:** Alexandra Lenhard, Monika Daseking

**Affiliations:** 1 Psychometrica, 97337 Dettelbach, Germany; 2 Department of Educational Psychology, Helmut-Schmidt-Universität/University of the Federal Armed Forces Hamburg, 22043 Hamburg, Germany

**Keywords:** school performance, IQ, *GAI*, *CPI*, school grades, linear regression, learning disorders

## Abstract

IQ scores are often used to predict school performance. However, for children with learning disabilities, the predictive validity of IQ scores appears to be low. In the fourth and fifth versions of the Wechsler Intelligence Scales for Children (WISC), new ancillary indices were introduced. The *General Ability Index* (*GAI*) is a broad measure of fluid reasoning with verbal, visual–spatial, and figural test items. By contrast, the *Cognitive Proficiency Index* (*CPI*) combines different executive functions known to be frequently affected in children with dyslexia, ADHD, or combined learning disorders. To date, there is little evidence to demonstrate that these measures improve the prediction of school performance beyond the *Full-scale IQ* (*FSIQ*). We therefore used lasso regression to explore the predictive validity of these measures for school grades. The analyzed samples were taken from the German standardization samples of the WISC-IV and the WISC-V. In most cases, the prediction of school performance was not considerably improved by taking the *GAI* or the *CPI* into account. However, when the individual discrepancy between the *CPI* and the *GAI* was high, the *FSIQ* lost its predictive validity in elementary school. In this subgroup of children, reading and writing skills were best predicted by the *CPI*, whereas math skills were limited by the lowest score (i.e., the minimum out of the *CPI* and the *GAI*).

## 1. Introduction

Results of intelligence tests are often used in childhood and adolescence to make decisions about the adequate type of school, grade level, or support measures. Moreover, the measured IQ is used as a predictor of school performance. In fact, IQ is a measure that has surprisingly high reliability and predictive validity compared to many other psychometric measures [1,2,3]. Nevertheless, there are also many individual cases where IQ fails as a predictive measure of school performance. For example, children with learning disorders often fall into this category. In fact, a striking discrepancy between perceived intelligence and the inability to learn to read and write was already reported for one of the earliest cases of dyslexia described in the scientific literature in 1896 [4]. The observation of such a discrepancy was even so remarkable that it has long been the basis for the diagnosis of learning disorders. While the DSM-5 [5] has now abandoned the ability–achievement discrepancy as a requirement for diagnosing learning disorders, the ICD-11 definition [6] still requires the affected academic skill to be markedly below what would be expected for general level of intellectual functioning. To put it differently, the individual IQ must by definition fail as a predictor of the academic skill in order for the learning disorder to be diagnosed. Moreover, adverse external conditions such as economic or environmental disadvantage, lack of instruction, or difficulties in speaking or understanding the language, which also affect school performance, are explicit exclusion criteria for the diagnosis of a learning disorders too [5,6].

Some cases of learning disorder might result from the fact that there are other individual factors besides intelligence that affect school performance. The most important personality factors to be mentioned here are motivation, emotion, and conscientiousness [7,8]. As a consequence, depending on the measures used, the average correlation between intelligence and school performance is only about *r* = 0.50 to 0.60 [3,9]. In many cases, however, no such exceptional personality traits (e.g., very low motivation or very high school anxiety) can be found either. In these cases, instead of the general level of cognitive functioning or other personal traits, an alternative explanation for the poor school performance could be the general heterogeneity of cognitive abilities in children with learning disorders [10]. In fact, in addition to the striking discrepancies between ability and school performance, children with learning disorders often exhibit exceptionally high intraindividual differences between other cognitive abilities too. For example, children with specific reading and/or writing disorder typically perform substantially below matched control groups on measures of working memory and/or perceptual speed, but not necessarily on measures of visual processing, fluid reasoning, or verbal comprehension [11,12,13,14]. For children with specific problems in mathematics, the evidence is a little bit more complex. Some studies showed that working memory is not only a specific predictor of reading and writing skills, but also a predictor of mathematical skills [15]. By contrast, others demonstrated that dyscalculia is typically accompanied by difficulties in fluid reasoning [2,16,17] and/or visual processing [2,18]. Since many cognitive abilities can be low in children with dyscalculia, it comes as no surprise that measures of general cognitive functioning (i.e., IQ) on average are also lower in these children compared to control groups [2] and IQ usually is a better predictor of mathematical skills than of reading and writing skills [3].

### 1.1. The Wechsler Intelligence Scales

Since the seminal work of Spearman in the early 20th century [19], it has been widely acknowledged that different cognitive abilities are substantially correlated with each other, indicating a latent general factor also referred to as “g”. However, there is still disagreement on how the different facets of intelligence relate to each other and how they are hierarchically structured. The most frequently used intelligence test today is the Wechsler Intelligence Scale for Children (WISC) [2]. Like other widespread intelligence tests [20,21], it is now predominantly aligned with the Cattell–Horn–Carroll (CHC) model of intelligence, the latter being supported by an overwhelming body of evidence [22]. The CHC model, which postulates three levels of varying specificity, contains more than 80 narrow cognitive abilities (e.g., quantitative reasoning, auditory short-term storage, retrieval fluency etc.) on level one, up to 17 broad abilities (e.g., fluid reasoning, working memory capacity, visual processing, etc.) on level two, and a general factor on level three. This general factor is predominantly inspired by Carroll’s three-stratum model of intelligence [1,23]. Carroll, however, was not the first to propose a three-stratum model. In fact, Alexander [24] already proposed a similar idea as early as 1935, stating that one common g-factor is not sufficient to explain the covariances between individual cognitive abilities, but that there are several other less general factors below the g-factor representing clusters of cognitive abilities. Wechsler recognized the importance of Alexander’s work shortly thereafter [25]. However, although he equally assumed that there were several such clusters, in his own intelligence tests, he grouped the subtests into only two broad scales: a *Verbal* and a *Performance* scale [26,27,28], noting that this was a content-based rather than a statistically verified grouping. Unfortunately, this second-level split far from corresponded to today’s differentiated model of intelligence, both in terms of the number and the content of the ability clusters. It was only after Wechsler’s death that the Wechsler scales were more closely adapted to the empirical findings that had since been established. The WISC-III [29] was the first edition of the Wechsler scales to offer four different indices below the level of the *Full-scale IQ* (*FSIQ*) that roughly corresponded to the broad abilities described today in the CHC model. These indices were called *Verbal Comprehension*, *Perceptual Organization*, *Freedom from Distractibility*, and *Perceptual Speed*. The *Perceptual Organization Index* represented aspects of both visual processing and fluid reasoning. The *Freedom From Distractibility Index* combined several broad abilities but had the highest emphasis on working memory. In fact, this scale was later revised and renamed as the *Working Memory Index* in the WISC-IV [30]. In addition, the fluid reasoning component was also increased in this edition of the test. The *Perceptual Organization Index* was therefore renamed as the *Perceptual Reasoning Index*. The fifth edition of the WISC [2] completed the shift towards the CHC model by splitting the *Perceptual Reasoning Index* into the *Visual Spatial Index* and the *Fluid Reasoning Index*.

Nevertheless, the five-factor structure of the WISC-V has repeatedly been subject to criticism. In particular, it was noted that, depending on the method and sample used, only one to four factors could be extracted from the subtests with factor analysis [31,32,33]. The development and critique of the Wechsler scales show that grouping subtests into broader scales is not a one-way street. Accordingly, the WISC-V does not only provide a five-factor structure. Alternatively, 9 out of 10 primary subtests can also be grouped into two broad ancillary indices, namely the *General Ability Index* (*GAI*) and the *Cognitive Proficiency Index* (*CPI*). This grouping is based on content as well as practical considerations in a manner similar to David Wechsler’s division of the subtests into a *verbal* and a *performance* scale. We will describe the content and possible use of the *GAI* and the *CPI* in more detail below.

#### 1.1.1. GAI

The *GAI* was first developed for use with the WISC–III to offer additional flexibility in the assessment of cognitive abilities [34]. The goal was to establish an index that is less sensitive to the influence of working memory and perceptual speed. Consequently, eight subtests from the *Verbal Comprehension Index* and the *Perceptual Organization Index* were combined in the original scale, whereas subtests explicitly measuring working memory or perceptual speed were excluded. The development of the *GAI* was largely driven by the diagnosis of learning disorders, which at that time mandated a discrepancy between ability and achievement, with the IQ score representing ability and tests on reading, writing, or mathematics representing achievement. Consequently, special education services were only granted when the IQ score was markedly higher than the results in the according achievement tests. At the same time, it was well known that many children with learning disabilities also have deficits in working memory and perceptual speed, which in turn decreased the *FSIQ* in the WISC [34]. Thus, calculating an ability index without working memory and perceptual speed increased the ability–achievement discrepancy and therefore the chance of receiving special education services if school performance was markedly below average.

Of course, the WISC has changed significantly from the third edition to the fifth edition. These changes have also affected the *GAI*, which is now calculated from only five subtests, namely similarities, vocabulary, block design, matrix reasoning, and figure weights. The subtests are drawn from the *Verbal Comprehension Index*, the *Visual Spatial Index*, or the *Fluid Reasoning Index* only. (The primary subtest, visual puzzles, from the *Visual Spatial Index*, is not included.) All of these subtests are characterized by high g-loadings, ranging from 0.67 to 0.72 [2]. The *GAI* still excludes subtests measuring working memory and perceptual speed and can therefore be interpreted as a broad scale of fluid reasoning containing verbal, figural, as well as quantitative content.

#### 1.1.2. CPI

The *CPI* is basically the counterpart to the *GAI* in that it combines all of the primary subtests of the WISC that measure either working memory or perceptual speed. In the WISC-V, the included subtests are digit span, picture span, symbol search, and coding. These subtests on average show much lower g-loadings than the *GAI* subtests, ranging from 0.36 to 0.65 [2]. The abilities measured in these scales are also often referred to by the term *executive functions* [35] and are closely related to attentional control [22,36,37]. It has to be noted, though, that the correlation between the *Working Memory Index* and the *Percepual Speed Index* is rather low (*r* = 0.36). Hence, at first glance, it seems that the subtests form the *Working Memory Index* and the *Perceptual Speed Index* should not be combined into another index beyond the *FSIQ*, since they appear to have little shared variance and almost all of it is already captured in the *FSIQ*. What makes the *CPI* interesting to practitioners nonetheless is that many clinical groups score exceptionally low on this specific index [38]. Specifically, they score below what would be expected based on the average *FSIQ* and the correlation between the *FSIQ* and the *CPI*. For example, the data of clinical groups presented in the WISC-V technical manual [2] indicate that this holds true for mildly and moderately disabled children, for children with attention deficit/hyperactivity disorder (ADHD), and for children diagnosed with autism spectrum disorder with or without language impairment. (Note that for gifted children, the *CPI* on average is not below what would be expected based on the *FSIQ*. Although it is well known that these children usually score lower on the *Working Memory Index* and the *Perceptual Speed Index* than on the *Verbal Comprehension Index*, the *Visual Spatial Index*, and the *Fluid Reasoning Index* [2,30], their average *CPI* is actually only within the range one would expect based on the regression toward the mean).

### 1.2. Rationale and Research Questions

Although the *GAI* is developed explicitly to diagnose learning disorders, its purpose is not to directly predict school performance. Instead, the stated goal is to maximize the ability–achievement discrepancy, which has long been considered an essential criterion of learning disorders. Since the *GAI* is supposed to represent ability in this procedure, the logical conclusion is that it cannot be a good predictor of academic skills. By contrast, it must be a particularly poor one. Otherwise, it may not maximize the discrepancy between ability and achievement.

On the one hand, this line of reasoning is misguided for two reasons. First, it has repeatedly been demonstrated that IQ scores combining different verbal and non-verbal subtests, such as the *FSIQ* in the WISC-V, are at least moderately good predictors of school performance [3]. Since the *GAI* correlates to *r* = 0.96 with the *FSIQ*, the same must hold true for the *GAI*. Second, if the *FSIQ* fails as a predictor of academic achievement for children with learning disorders, then this should hold true for the *GAI* too. The question then is if the *GAI* should be used at all to diagnose learning disorders. In fact, the requirement of an ability–achievement discrepancy in the diagnosis of learning disorders has at least been removed from the DSM.

On the other hand, it is consistent with both our practical experience and empirical data that children with low school performance nevertheless often show exceptionally large discrepancies between general measures of cognitive functioning and school performance [11,12,13,14,15,16,17]. We therefore propose a more direct and parsimonious explanation for the observed discrepancies, namely that they are not an indicator of the learning disorders themselves. Instead, they can simply be interpreted as an indicator of an unusually high heterogeneity among the individual cognitive abilities. Note that academic skills such as reading, writing, and mathematical skills are perceived as broad cognitive abilities in the CHC model. Thus, if these abilities deviate considerably from measures such as the *FSIQ*, they must also deviate from at least some of the broad cognitive abilities that are captured in the *FSIQ*.

Furthermore, we assume that the *FSIQ* usually is a good predictor of school performance, but that it fails when heterogeneity among cognitive abilities is exceptionally high. Although practitioners have long relied on the assertion that the validity of global scores of intellectual functioning is diminished when heterogeneity across broad cognitive abilities is exceptionally high [39], to the best of our knowledge, this assertion has never been directly tested. Nevertheless, the assertion is highly plausible because the basis for calculating the *FSIQ* is the assumption that all cognitive abilities share a substantial amount of common variance. Hence, a specific cognitive ability should likely be low if the *FSIQ* is also low. Nevertheless, apparently, for some children, the heterogeneity among their cognitive abilities is much higher than expected on the basis of representative samples. Thus, this group of children exhibits considerably less common variance in their cognitive abilities. Therefore, it can be assumed that for these children, scales such as the *FSIQ* or the *GAI*, representing this shared variance, generally have low predictive validity for specific cognitive abilities, especially if these specific cognitive abilities have relatively low g-loading, such as basic reading and writing skills.

In this article, we wanted to analyze whether an exceptionally large discrepancy between the *GAI* and the *CPI* is an easy-to-handle indicator of low predictive validity of the *FSIQ*. To this end, we compared German-speaking children with and without an exceptionally high discrepancy between the *GAI* and the *CPI* with regard to the correlations between the *FSIQ* and school grades in mathematics and German. As is presumably also the case in other languages/countries, the curriculum in German as a school subject mainly focuses on basic reading and spelling skills in elementary school. In secondary school, it covers a much broader range of language skills, with an emphasis on advanced language and text comprehension as well as writing skills. We hypothesized that when the discrepancy between the *GAI* and the *CPI* is high, the correlations between the *FSIQ* and school grades in mathematics and German are significantly lower than when the discrepancy is low. Moreover, we assumed that this effect would be even larger for the grades in German than for the grades in mathematics because, as described above, mathematical skills seem to depend equally on fluid reasoning and working memory.

Since it has repeatedly been demonstrated that the predictive validity of IQ scores is generally lower in elementary school than in secondary school [3,40] (note that elementary school in Germany usually covers only the first four years of school, beginning at the age of six), we hypothesized that the predictive validity of IQ scores might also be more susceptible to effects of heterogeneity in elementary school than in secondary school. We therefore conducted all analyses separately for children in elementary vs. secondary schools.

If the *FSIQ* is indeed a poor predictor of school performance for children with exceptionally large discrepancy between the *GAI* and the *CPI*, then the question immediately arises as to which is the best predictor of school performance in this particular group. Specifically, the *CPI* might be a better predictor of reading and writing because the importance of working memory and perceptual speed for reading and writing has repeatedly been demonstrated [12,13,14]. Moreover, because the *CPI* captures processes closely related to attentional control [22,35,36,37], it may be particularly predictive in elementary school when automation with regard to basic academic skills, such as reading fluency and spelling, is still low. Mathematical skills, on the other hand, seem to correlate more strongly with IQs than reading and writing skills. Therefore, despite reduced predictive validity, the *FSIQ* might still be the best predictor of mathematical skills, even if the discrepancy between the *GAI* and the *CPI* is exceptionally high.

However, there is also another possible explanation as to why the *FSIQ* might be a bad predictor in the case of heterogeneous intraindividual cognitive abilities. The predictive validity of the *FSIQ* is based on the assumption of a compensation model. Such models assume that poor performance in one cognitive ability can at least partially be compensated by good performance in the other abilities. However, school performance of children with high heterogeneity in their cognitive abilities might be better explained by a deficit model. In deficit models, the overall performance is limited by the lowest single determinant. To explore the questions described above, we additionally modelled the data with lasso regression (least absolute shrinkage and selection operator regression).

## 2. Materials and Methods

### 2.1. Sample

Since from the statistical point of view, exceptionality is defined by 5 to 10% of the most extreme cases, and large samples are needed to gather enough exceptional cases for statistical analysis. Therefore, we used two large standardization samples of the German adaptations of the WISC-IV [41] and the WISC-V [42]. For some of these children, school grades in mathematics and German were collected as part of validation studies. The WISC-IV sample comprised *n* = 1745 children, for whom school grades in German and mathematics were available. These data were gathered between March 2005 and June 2006. For the WISC-V, *n* = 825 complete datasets were available, collected between September 2015 and February 2017.

The data were collected in Germany (*n* = 2306), Austria (*n* = 121, WISC-IV only), and Switzerland (*n* = 143, WISC-IV only), and included *n* = 1293 male and *n* = 1277 female children, with *n* = 686 children attending elementary school and *n* = 1884 attending secondary school. In elementary school, age ranged from 6.27 to 12.88 years (*M* = 9.32; *SD* = 1.10), with most children being between 8 and 10 years old. Only 36 children were younger than 8 and 35 were older than 10. In secondary school, age ranged from 9.12 to 16.99 years (*M* = 13.69; *SD* = 1.83). Overall, 19.6% of all children came from a household where at least one parent was not a native German speaker. In the WISC-IV sample, this proportion was only 15.1%, compared to 29.1% in the WISC-V sample, with different proportions reflecting the respective census data at the time of the data collection. Children attending special schools were generally not included in the analyses, because the grades at these schools were usually difficult to compare with grades at other schools. However, only 3.2% of all children of the original samples attended such schools. Apart from that, the total sample can be considered nearly representative.

Table 1 shows the sample size as a function of age. As can be seen, the subsample sizes for ages 6 to 8 were lower compared to all other age levels. This is because usually children in Germany do not usually receive grades during the first two years of school.

### 2.2. Measures

#### 2.2.1. Grades

The grades in Germany, Austria, and Switzerland ranged from 1 to 6, with 1 and 6 being the best and worst grade, respectively, in Germany and Austria, whereas in Switzerland it was exactly the opposite. Therefore, all grades collected in Switzerland were recoded to match the system of the other two countries.

#### 2.2.2. FSIQ

The *FSIQ* was determined for both the WISC-IV and the WISC-V, as described in the respective manuals, that is, in the case of the WISC-IV, the index was derived from 10 different subtests, whereas in the case of the WISC-V, only 7 subtests were used. Despite the different number of subtests, the correlation between the corresponding *FSIQ* scores of both instruments (corrected for the variability of the normative sample) is *r* = 0.89 [42], which is almost as high as the test–retest correlation of the German WISC-V (*r*_tt_ = 0.90). The *FSIQ* scores of the WISC-IV and the WISC-V can therefore be regarded as almost equivalent.

#### 2.2.3. GAI

In the WISC-V, the *GAI* is derived from five different subtests, namely similarities, vocabulary, block design, matrix reasoning, and figure weights. These subtests are drawn from the *Verbal Comprehension Index*, the *Visual Spatial Index*, or the *Fluid Reasoning Index*. Unfortunately, neither index scores for the *GAI* nor for the *CPI* were provided in the original German adaptation of the WISC-IV. It was only several month later that index scores were made available for the *GAI* [43]. Their calculation was based on the guidelines proposed in the “Essentials of WISC-IV Assessment” [44]; thus, they were derived from three verbal comprehension subtests (i.e., vocabulary, comprehension, and similarities) and three *perceptual reasoning subtests* (i.e., block design, matrix reasoning, and picture concepts). More precisely, the *Verbal Comprehension Index* and the *Perceptual Reasoning Index* were added up to derive the *GAI*. In this article, however, we slightly deviated from this calculation because we wanted to derive an index for the WISC-IV that is as similar as possible to the WISC-V *GAI*. We therefore dropped the comprehension subtest, which is no longer a primary subtest in the WISC-V and is not included in the *GAI* either. All other subtests were retained. Note that we also had to keep the picture concepts subtest, because the figure weights subtest did not yet exist. Hence, for all children in the WISC-IV sample, we added up the scaled scores of similarities, vocabulary, block design, matrix reasoning, and picture concepts. Subsequently, we performed inverse normal transformation to transform the sum to normal scores. Finally, the normal scores were converted to index scores by multiplying each score by the standard deviation of 15 and adding the mean score of 100.

The reliability coefficient of the WISC-V *GAI*, as indicated in the manual of the German adaptation, is *r*_tt_ = 0.95. For the WISC-IV, we estimated the overall reliability of the *GAI* from the reliability coefficients of the five subtests using the formula of Nunnally and Bernstein [45], i.e., *r*_tt_ = 0.94.

#### 2.2.4. CPI

In the WISC-V, the *CPI* is derived from digit span, picture span, symbol search, and coding. Since picture span from the *Working Memory Index* did not yet exist in the WISC-IV, we instead used letter–number sequencing to calculate the *CPI* in the WISC-IV sample. This subtest served as the second subtest of the *Working Memory Index* in the WISC-IV. Hence, for all children from the WISC-IV sample, we added up the scaled scores of digit span, letter–number sequencing, symbol search, and coding. The subsequent transformation into index scores was performed in the same way as for the *GAI*. The manual of the German WISC-V reports a reliability coefficient of *r*_tt_ = 0.93 for the *CPI*. For the WISC-IV, we again estimated its overall reliability from the reliability coefficients of the subtests using the formula of Nunnally and Bernstein [45]. The result was an overall reliability of *r*_tt_ = 0.91.

### 2.3. Grouping

We assigned the children to two different groups based on their individual difference between the *GAI* and the *CPI*. The first group consisted of the 10% most extreme cases and the second group consisted of all remaining children. To this end, we first calculated the difference between the *GAI* and the *CPI*. We subsequently determined the 10% most extreme cases (5% on each tail of the distribution). This group included a total of 263 children (131 with *GAI* − *CPI* ≤ −24 and 132 with *GAI* − *CPI* ≥ 23). Of these children, 64 attended elementary school and 199 attended secondary school. The remaining 2307 children were assigned to the second group of children with normal discrepancies between the *GAI* and the *CPI*. In this group, 623 attended elementary school and 1684 attended secondary school.

### 2.4. Data Analysis

#### 2.4.1. Comparison of Correlations

To determine whether the *FSIQ* predicted school performance significantly worse when the discrepancy between the *GAI* and the *CPI* was extremely large, we first calculated the correlations between the *FSIQ* and school grades. We performed this separately with regard to discrepancy (normal vs. large), subject (mathematics vs. German), as well as the type of school (elementary school vs. secondary school). Subsequently, we compared the correlations of children with large discrepancies between the *GAI* and the *CPI* to the correlations of children with normal discrepancies. To this end, we applied the statistical procedure described in [46]. The one-sided significance level was set to α = 0.05.

#### 2.4.2. Lasso Regression

We also used lasso regression to further explore the data. In order to identify the best predictor of school performance, we entered the *GAI*, the *CPI*, and the *FSIQ* as independent variables in the regression. Furthermore, to test whether deficit models have higher predictive validity than compensation models, at least for children with large discrepancies between the *GAI* and the *CPI*, we additionally included the minimum out of the potential predictors as an independent variable. The school grades in mathematics and German served as separate dependent variables in the general linear models. Again, we modelled the data separately with regard to discrepancies between the *GAI* and the *CPI* (normal vs. large), the subject (mathematics vs. German), and the type of school (elementary school vs. secondary school).

Lasso regression is particularly suited for these analyses because in the case of several correlated predictors, it picks the best predictor while “punishing” the others [47]. Moreover, the glmnet package we used for modeling the data on the R platform [48] includes k-fold cross-validation as a standard procedure. Therefore, the package returns parsimonious and stable linear models.

The regression parameter α was set to 1 for lasso regression. The number of folds for the k-fold cross-validation was set to 10.

The glmnet package additionally includes a complexity parameter λ with small parameters, leading to a higher number of predictors. To avoid overfit, the authors of the package recommend not using the λ, which achieves the smallest mean squared error (*MSE)* in the cross-validation (i.e., the best fitting model). Instead, the default was set to the largest λ, whereby the *MSE* in the cross-validation was within one standard error of the smallest *MSE* (λ_1*SE*). However, in some cases, this default setting returned no predictor at all. This was especially the case for the relatively small group of children with large discrepancies between the *GAI* and the *CPI*. In these cases, we used the largest λ providing at least one predictor. In all of these cases, the selected λ was only slightly smaller than λ_1*SE*.

## 3. Results

### 3.1. Comparison of Correlations

Table 2 contains the correlations between the different predictors (the *FSIQ*, the *GAI*, or the *CPI*) and school grades as a function of discrepancy between the *GAI* and the *CPI* (normal vs. large), the subject (mathematics vs. German), and the type of school (elementary school vs. secondary school).

First of all, as expected, all correlations between the *FSIQ* and grades were significantly negative (note that “1” represents the best grade in Germany and “6” represents the worst grade) regardless of the subject, type of school, or discrepancy between the *GAI* and the *CPI*, indicating that the *FSIQ* was in fact a significant predictor of school performance in all cases.

However, the correlations varied in magnitude. In elementary school, the correlations between the *FSIQ* and grades tended to be higher for children with normal discrepancies between the *GAI* and the *CPI* than for children with large discrepancies. This was true both for the grades in mathematics (*z* = −1.639, *p* = 0.051) and in German (*z* = −1.636, *p* = 0.051). Although both effects missed the 5% significance criterion by a very narrow margin, it becomes clear that they nevertheless should not be neglected when looking at the differences between both groups in terms of explained variance. In mathematics, the *FSIQ* explained more than twice as much variance in the grades for children with normal discrepancies than for children with high discrepancies (25.5% vs. 10.5%); in German, it was even more than three times as much variance (20.2% vs. 6.7%).

In secondary school, the correlations for children with normal discrepancy were slightly higher than the correlations for children with large discrepancy in numerical terms, but the differences between the correlations fell far short of significance in both cases (mathematics: *z* = −0.122, *p* = 0.451; German: *z* = −0.462, *p* = 0.322).

Unlike the *FSIQ*, the *GAI* and the *CPI* did not consistently predict school performance. When the discrepancy between the *GAI* and the *CPI* was within a normal range, all correlations of each of these scales with school grades were significant, but when it was exceptionally large, some of them were not. Most notably, for children with large discrepancies, the *GAI* only predicted school performance in secondary school but completely failed as a predictor in elementary school. By contrast, the *CPI* was at least a moderately good predictor in elementary school but almost completely lost its predictive validity in secondary school when the discrepancy between the *GAI* and the *CPI* was large.

### 3.2. Lasso Regression

To find optimal prediction models of school grades for the different groups (normal vs. large discrepancies between the *GAI* and the *CPI*), the types of school (elementary school vs. secondary school), and the subjects (mathematics vs. German), we performed lasso regression with the *GAI*, the *CPI*, the *FSIQ*, and the minimum out of these predictor scores. The results of these analyses are listed in Table 3.

Overall, the models explained much more variance in elementary school than in secondary school (22.5% vs. 13.8%; for calculating the variance proportions, the subsamples were weighted according to their size). They explained slightly more variance in the math grade as compared to the German grade (17.6% vs. 14.6%). Furthermore, they explained slightly more variance when the discrepancy between the *GAI* and the *CPI* was normal as compared to large (16.4% vs. 13.2%).

Interestingly, the *GAI* only occurred as a significant predictor of mathematics but never of German, whereas the *CPI* was more important as a predictor of German compared to mathematics.

However, most importantly, the *GAI*, the *CPI*, and the minimum score as predictors of school performance did not considerably increase the *R*-squared values (as compared to prediction with the *FSIQ* only) in three out of four subsamples. There was only one subsample in which the explained variance was considerably increased, which was the group of children in elementary school with a large discrepancy between the *GAI* and the *CPI*. In this group, the prediction of school performance was improved both for the grades in mathematics and German.

Only one predictor was selected by lasso regression when the German grade was the dependent variable, and this predictor was the *CPI*, not the *FSIQ*. The explained variance was increased from 6.7% (prediction with the *FSIQ* only) to 13.2%; thus, it was almost doubled. The gain of explained variance corresponds to a medium effect size of *d* = 0.53 [49]. When the regression parameter lambda was slightly reduced to force more predictors into the linear model, the minimum score was additionally selected as the second most significant predictor. However, its weight was less than one-sixth that of the *CPI* and therefore the inclusion of the minimum score as a second predictor did not significantly increase the *R*-squared values.

In mathematics, the *CPI* was also selected as a significant predictor, but the predictor with the highest weight was the minimum score. Its weight was even twice that of the *CPI*. The explained variance increased from 10.5% (prediction with the *FSIQ* only) to 18.6%, which also corresponds to a medium effect size of *d* = 0.59 [49].

## 4. Discussion

To determine whether the predictive validity of the *FSIQ* for school performance depends on the homogeneity of the test results in the WISC and to find optimal prediction models, we analyzed the significance of the *FSIQ*, the *GAI*, the *CPI*, or the minimum out of these variables as predictors of grades in mathematics and German as a function of the discrepancy between the *GAI* and the *CPI*. We performed this separately for children in elementary vs. secondary school.

The analysis of the correlations between the different scores and school grades demonstrated that the *FSIQ* alone was a significant predictor of school performance in all cases, regardless of the subject (mathematics vs. German), the type of school (elementary school vs. secondary school), or the discrepancy between the *GAI* and the *CPI*. However, for children in elementary school, a large discrepancy between the *GAI* and the *CPI* tended to decrease the predictive validity of the *FSIQ*. The significance level was missed quite narrowly, but the difference in terms of explained variance was so large that, in our opinion, it cannot be ignored in terms of its practical implications. Moreover, the explained variance in school grades was almost twice as high when predictors other than the *FSIQ* were used.

For the school grades in German, the *CPI* turned out to be the best predictor in this specific group. This result highlights the importance of the cognitive abilities measured in the *CPI* (i.e., working memory and perceptual speed) for the acquisition of basic reading and writing skills. The importance of working memory—especially auditory working memory—for these skills has repeatedly been shown in scientific literature [13,14]. Perceptual speed appears to play a more indirect role, mediated by attentional control, specifically shifting, and inhibition [35]. In addition, fine motor skills are also closely related to perceptual speed and could play a specific role in writing skills in elementary school. For example, it has been shown that children with dyslexia not only have a deficit in spelling but many of them also show difficulties in handwriting [50]. However, the etiology of such comorbidities is not entirely clear. On the one hand, reduced perceptual speed could have a direct causal effect on both the development of fine motor skills and spelling. On the other hand, deficits in fine motor skills could also divert attentional resources from other tasks such as spelling.

Unfortunately, our sample contained only few 6- and 7-year-old children. The reason for this is that school grades in Germany are usually not assigned before the third-grade level. However, we do not believe that this shortcoming calls into question the basic results. On the contrary, it can be assumed that the influence of attentional control is even more pronounced in the first two years of school because the automation of basic academic skills is still extremely low at this early stage.

In line with this assumption, the predictive validity of the *CPI* for language skills seems to decrease in secondary school, that is, when most of the processes involved in basic reading and writing skills have been automated [9,51] and can therefore be performed without attentional control. In addition, in secondary school, the native language curriculum focuses much more on complex tasks, such as text comprehension and interpretation, than on basic reading and writing skills. Therefore, the role of verbal comprehension and fluid reasoning may be more important in secondary school compared to elementary school. Importantly, however, there were only weak indications that deficit models would markedly improve the prediction of the language skills in secondary school as compared to compensation models. Hence, the *FSIQ* seems to be a valid predictor of language skills in secondary school, even if the discrepancy between the *GAI* and the *CPI* is large.

In mathematics, the pattern was somewhat different. When the discrepancy between the *GAI* and the *CPI* was large in elementary school, the most important predictor of the grades was the minimum out of the *GAI* and the *CPI*. (Note that the *FSIQ* is never the minimum score when the discrepancy between the *GAI* and the *CPI* is large.) This result suggests that for children with large intraindividual heterogeneity between different cognitive domains, compensation models provide suboptimal results with regard to the prediction of mathematical skills, at least in elementary school. As mentioned in the introduction, mathematical skills generally correlate more strongly with intelligence than reading and writing. In addition, children with dyscalculia often show intraindividual weaknesses in visual processing and fluid reasoning, but some of them also show intraindividual weaknesses in working memory and/or perceptual speed. The latter result already indicates that individual deficits in determinants of mathematical ability can have a limiting effect on the overall math performance. The results we obtained with lasso regression confirm this assumption. Interestingly, in secondary school, lasso regression also favored a minimum score over the *FSIQ* as a significant predictor of grades in mathematics, regardless of the discrepancy between the *GAI* and the *CPI*. Admittedly, though, the prediction of math skills was only slightly improved when using a deficit model as compared to prediction with the *FSIQ* only. Therefore, the *FSIQ* will probably be a valid, reliable, and easy-to-use predictor in secondary school in most cases, regardless of the subject or heterogeneity of the cognitive abilities. One must not forget, though, that the minimum score is highly correlated with the *FSIQ*, with correlations above *r* = 0.90 in every single subgroup. However, this is exactly why the advantage of deficit models presumably grows with the discrepancy between the *FSIQ* and the minimum score.

In summary, we demonstrated that, at least in elementary school, the *GAI* and the *CPI* can be used to markedly improve the prediction of school performance compared to the use of the *FSIQ* only. Specifically, a large discrepancy between both indices indicates a loss of predictive validity in the *FSIQ*. Of course, the question arises why one should use atheoretical scales such as the *GAI* and the *CPI* for prediction purposes in the first place, when the construct validity of the five primary indices used in the WISC-V is empirically established. Unfortunately, we could not reliably test the prediction of school performance with these five indices. Very large samples are needed to examine children with exceptionally large discrepancies between the cognitive abilities, which was the reason for compiling the German standardization samples of both the WISC-IV and the WISC-V. However, there are relatively large differences between the primary scales in the WISC-IV and the WISC-V, with the WISC-IV only providing four instead of five index scales. We therefore leave this issue to future studies that may be conducted with the compiled standardization samples of the WISC-V and the WISC-VI.

Although we faced a methodological barrier here, there are some theoretical and practical reasons for using the *GAI* and the *CPI* instead of the five primary indices. First, the *GAI* and the *CPI* are, on average, more reliable than the primary indices because they are each based on more subtests. Second, two scales can be compared faster and easier to each other than five different indices (simple difference score vs. complex profile analysis). Apart from these aspects, the same caveat that applies to the *FSIQ* presumably also applies to the two scales: the validity of the scales might be markedly decreased in case of too much heterogeneity within each scale. To put it differently, using the five primary indices probably does not significantly improve the prediction of academic achievement when intraindividual heterogeneity within the *GAI* and the *CPI* is small, but it might do so when heterogeneity is large. The use of the scales thus requires that homogeneity within the scales is ascertained beforehand.

Unfortunately, there were also a few limitations which restrict the validity of our results. First of all, the WISC-IV and the WISC-V contain different subtests, which is why the *GAI* and the *CPI* were derived in slightly different ways in the two subsamples. Specifically, we used two auditory working memory subtests to derive the *CPI* in the WISC-IV subsample, whereas in the WISC-V, one auditory and one visual–spatial working memory subtest enter the *CPI*. This difference might be important since auditory working memory has been shown to be more important for reading and writing skills as compared to visual–spatial working memory [13,52]. Therefore, the ancillary auditory working memory scale of the WISC-V might have a higher predictive validity than the *CPI*. However, there was no indication in our data that this is in fact the case.

Second, the *FSIQ* is derived from a different number of subtests in the two different WISC versions. While all ten core subtests are used in the WISC-IV, “only” seven of the ten primary subtests are used in the WISC-V. However, since the correlation between the corresponding *FSIQ* scores of both instruments is almost as high as the test–retest correlation of the German WISC-V, we do not think that the different number of subtests plays a major role.

Third, the school system in Germany is such that after elementary school—which in Germany usually covers only the first four years of school—children are assigned to different school types based on their previous school performance. This system leads to limited variance in the cognitive abilities of each individual type of school. Furthermore, the same grade does not necessarily reflect the same level of proficiency when achieved in different types of schools. Both factors lead to a reduced covariation in the secondary school variables. Therefore, the validity of the models and results is probably markedly lower in secondary school as compared to elementary school.

Fourth, we used grades as dependent variables. They can be easily collected and were therefore collected by default in the WISC-IV and WISC-V standardization. Nevertheless, they have certain drawbacks too. First, the curriculum in German (as presumably in other countries too) mainly focuses on basic reading and writing skills in elementary school but covers a wide range of linguistic skills in secondary school, for example, knowledge about different types of texts, literary periods, or authors. Therefore, the grade in German measures very different skills and abilities in elementary school as compared to secondary school. Second, grades usually are not normally distributed. Third, their variance is smaller than that of standardized achievement tests. Fourth, they tend to be less reliable than the results of standardized achievement tests because they are partly based on subjective judgments and are therefore susceptible to various types of judgment biases. Therefore, the variance explained by the different models was certainly less than it would have been if standardized achievement tests were used as dependent variables.

Finally, it is well known that gifted children frequently score much lower on the *CPI* as compared to the *GAI*. By contrast, the cognitive profiles of children with a below-average IQ or intellectual disability are much more homogeneous on average [2,30]. Hence, whether a certain discrepancy between the *GAI* and the *CPI* is actually exceptional or not also depends on the general ability level of the child. The results obtained in this study may therefore not apply equally to all levels of general cognitive ability. However, methodologically, it is extremely difficult and costly to include the general ability level as a qualifying factor in such studies, as this would require extremely large samples. In particular, the question remains as to whether the abilities captured in the *CPI* are a limiting factor for the acquisition of basic academic skills in gifted children to the same extent as in children with an average or a below-average IQ score.

## 5. Conclusions

Decisions about school placement or support measures are often based solely on IQ scores. Our analyses have shown that increased attention also needs to be paid to heterogeneity in the intraindividual cognitive profile, especially in elementary school. Unfortunately, the ICD-11 still requires academic performance to be below what would be expected for chronological age and level of intellectual functioning in order to diagnose learning disorders. To put it differently, a lack of predictive validity in the IQ score is considered a necessary criterion of the disorder. Clearly, we showed that the predictive validity of the *FSIQ* only decreases when the discrepancy between the *CPI* and the *GAI* is exceptionally large. Hence, the affected children were in fact exceptional in certain respects. However, exceptionality in and of itself is not necessarily a sign of pathology; otherwise, giftedness would also have to be considered a disorder. In our opinion, it is the low performance that makes the learning disorder a disorder, not the fact that existing (and probably insufficient) predictive models fail. After all, no one would argue that cancer is a disease and should be treated only if it was not expected for chronological age and level of physiological functioning, or that a hurricane is only bad weather if the weather forecast was wrong. The only difference is that the necessary actions can be taken earlier if the forecast is correct.

In light of the findings described in this article, we consider it imperative that the ability–achievement discrepancy criterion, which has long since been removed from the DSM [5], will finally be removed from future versions of the ICD as well. As shown, the expectation for academic achievement may turn out differently if intraindividual heterogeneity in the cognitive abilities is taken into account in the prediction, at least in elementary school. Most importantly, the prediction of academic achievement can be improved if exceptionally high intraindividual deficits, such as weak working memory and attentional control, are included as predictors instead of IQ alone.

Finally, our data have shown that cognitive measures are generally limited predictors of school performance even when heterogeneity is included. However, whether and how other factors, such as conscientiousness, motivation, or socioeconomic status, should be included in school decisions goes beyond the scope of this article because such data were not contained in our samples.

## Figures and Tables

**Table 1:** Sample size as a function of age.

Age	Sample Size
6	18
7	18
8	164
9	275
10	243
11	332
12	325
13	298
14	297
15	301
16	251
Total	2570

**Table 2:** Correlations between possible predictors (the *FSIQ*, the *GAI*, or the *CPI*) and grades in German and mathematics as function of discrepancy between the *GAI* and the *CPI* and the type of school.

Discrepancy between the *GAI* and the *CPI*		Elementary School		Secondary School	
		Math Grade	German Grade	Math Grade	German Grade
Normal	Correlation with the *FSIQ*	−0.505 **	−0.449 **	−0.370 **	−0.348 **
	Sig. (two-sided)	0.000	0.000	0.000	0.000
	*n*	623	623	1684	1684
	Correlation with the *GAI*	−0.502 **	−0.419 **	−0.378 **	−0.319 **
	Sig. (two-sided)	0.000	0.000	0.000	0.000
	*n*	623	623	1684	1684
	Correlation with the *CPI*	−0.403 **	−0.402 **	−0.322 **	−0.351 **
	Sig. (two-sided)	0.000	0.000	0.000	0.000
	*n*	623	623	1684	1684
Large	Correlation with the *FSIQ*	−0.324 **	−0.258 *	−0.362 **	−0.317 **
	Sig. (two-sided)	0.009	0.039	0.000	0.000
	*n*	0 64	64	199	199
	Correlation with the *GAI*	−0.087	−0.055	−0.370 **	−0.226 **
	Sig. (two-sided)	0.495	0.669	0.000	0.001
	*n*	64	64	199	199
	Correlation with the *CPI*	−0.382 **	−0.363 **	−0.005	−0.164 *
	Sig. (two-sided)	0.002	0.003	0.943	0.021
	*n*	64	64	199	199

* Correlation significant with *p* < 0.05 (two-sided), ** correlation significant with *p* < 0.01 (two-sided).

**Table 3:** Regression models to predict grades in German and mathematics from the *GAI*, the *CPI*, the *FSIQ*, and the minimum out of these three predictors.

Discrepancy between the *GAI* and the *CPI*	Type of School	*n*	Subject	*R* Squared ^a^ (comp. to *FSIQ* Only)	Unstandardized Weights				
					Intercept	*GAI*	*CPI*	*FSIQ*	MIN ^b^
Normal	Elementary School	623	Math	0.261 (+0.006)	4.35	−8.75 × 10^−3^		−1.30 × 10^−2^	
			German	0.202 (+0.000)	3.96			−1.73 × 10^−2^	
	Secondary School	1684	Math	0.147 (+0.011)	4.46	−1.08 × 10^−2^			−7.06 × 10^−3^
			German	0.132 (+0.011)	4.10		−5.72 ×10^−3^	−6.89 × 10^−4^	−7.50 × 10^−3^
Large	Elementary School	64	Math	0.186 (+0.081)	2.40		−1.07 ×10^−3^		−2.28 × 10^−3^
			German	0.132 (+0.065)	2.51		−1.97 ×10^−3^		
	Secondary School	199	Math	0.147 (+0.016)	3.38	−4.80 × 10^−3^		−5.94 × 10^−4^	−1.85 × 10^−3^
			German	0.100 (+0.000)	2.86			−1.68 × 10^−3^	

^a^*R*-squared values were computed for the whole subsample (not only the cross-validation groups). The value in parentheses indicates the increase in *R*-squared values as compared to prediction with the *FSIQ* only (note that it is not necessary to specify an adjusted *R*-squared value when using lasso regression, since overfitting is already controlled by k-fold cross-validation). ^b^ MIN = minimum out of the *GAI*, the *CPI*, and the *FSIQ.*

## Data Availability

Since the data originate from copyrighted psychometric tests, there is unfortunately no possibility to make these data publicly available.
